# Improved dsRNA isolation and purification method validated by viral dsRNA detection using novel primers in *Saccharomyces cerevisiae*

**DOI:** 10.1016/j.mex.2023.102435

**Published:** 2023-10-11

**Authors:** Fernando M.H. Cardoso, Alexandre Elias, Inês Pereira, Isabel Maurício, Olga Matos

**Affiliations:** a Global Health and Tropical Medicine, GHTM, Associate Laboratory in Translation and Innovation Towards Global Health, LA-REAL, Instituto de Higiene e Medicina Tropical, IHMT, Universidade NOVA de Lisboa, UNL, Rua da Junqueira 100, Lisboa 1349-008, Portugal; b Instituto Gulbenkian de Ciência, Oeiras 2780-156, Portugal; c Environmental Health Institute, Faculdade de Medicina da Universidade de Lisboa, Lisboa 1649-028, Portugal

**Keywords:** Improved dsRNA isolation and purification- phase partition, Viral dsRNA, Phenol extraction, Ammonium sulphate, RT-PCR, Sequencing

## Abstract

Accurate genomic sequencing demands high-quality double-stranded RNA (dsRNA). Existing methods for dsRNA extraction from yeast, fungi, and plants primarily rely on cellulose, suitable only for small volume extractions, or the time-consuming lithium chloride precipitation. To streamline the traditional phenol-chloroform-based dsRNA extraction method, the main challenge is the reduction of mitochondrial DNA (mtDNA) and Single Stranded RNA (ssRNA) to no detectable levels after gel electrophoresis. This challenge is successfully addressed through the modified approach described here, involving phenol extraction at low pH, followed by the addition of ammonium sulfate to the aqueous buffer. The dsRNA isolated using this novel method exhibits comparable quality to that obtained through cellulose purification, and it is readily amenable to RT-PCR. Moreover, a single batch of yeast cell RNA isolation requires only 2-3 h of hands-on time, thus simplifying and expediting the process significantly.

• Buffers were redesigned from [32,^33^,35].
• No DNASE, Ribonuclease A or beads were used during the purification.
• Simple and inexpensive dsRNA extraction and purification method is described.

## Graphical abstract

**Figure:**
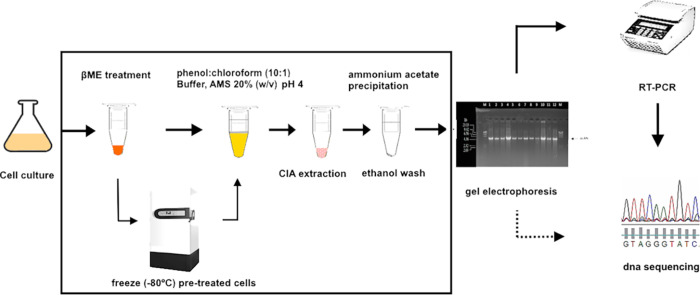

Specifications table

**Table:** 

Subject area:	Biochemistry, Genetics and Molecular Biology
More specific subject area:	dsRNA isolation and purification
Name of your method:	Improved dsRNA isolation and purification- phase partition
Name and reference of original method:	[1] Fried HM, Fink GR. Electron microscopic heteroduplex analysis of “killer” double-stranded RNA species from yeast. Proc Natl Acad Sci U S A. 1978;75(9):4224-4228. 10.1073/pnas.75.9.4224 [2] Flegr J. A rapid method for isolation of double stranded RNA. Prep Biochem. 1987;17(4):423-433. 10.1080/00327488708062505 [3] Knapp JE, Chandlee JM. RNA/DNA mini-prep from a single sample of orchid tissue. Biotechniques. 1996;21(1):54-56. 10.2144/96211bm11
Resource availability:	Viral RNA-directed RNA-polymerase from L-BC Totivirus gene sequence (NCBI Submission ID: OR393309)

## Method details

### Overview

Double-stranded RNA (dsRNA) is a crucial molecule involved in various biological processes, including RNA interference [1], interferon [2,3], viral replication [4], [5], [6], [7] or endogenous dsRNA [8,9]. Viruses that have been found in the economically important species *Saccharomyces cerevisiae* belong to the groups Partitivirus [10], Totivirus [11], and Narnavirus [12], which includes as *Saccharomyces* 20S and 23S RNA [13]. All these viruses contain a dsRNA genome.

DsRNA isolation is, therefore, an essential step to study these processes and to identify dsRNA viruses. The ability to isolate dsRNA is a valuable tool to investigate the viral diversity and evolution [14].

The most used methods of dsRNA isolation use fibrous or microgranular cellulose. These methods rely on the affinity of cellulose powder specifically for nucleic acids, followed by dsRNA adsorption with an ethanol concentration of 15 %, and then, release of the dsRNA from cellulose without ethanol [15], [16], [17], [18], [19], [20], [21]. Other methods use Hydroxyapatite alone [22], [23], [24] or in combination with cellulose [25], differential precipitation of ssRNA and dsRNA with Lithium chloride [26] or various other types of resins [27], [28], [29], [30]. In these methods, an initial nucleic acid extraction is done, mainly with phenol (pH 7), followed by chromatographic separation to obtain dsRNA. All these methods are time-consuming or expensive.

Furthermore, for the initial cell lysis step these methods generally used enzymes or beads [31], which can be expensive or require specialized equipment, respectively. An alternative method for cell pre-treatment, consists of washing with EDTA, followed by an incubation in Tris-H_2_SO_4_ buffer with 2-mercaptoethanol (βME) before phenol extraction [32].

Nucleic acids can be partitioned between the aqueous or the phenol phase according to the pH. For example, phenol equilibrated with a pH 4 buffer can be used to remove DNA from the aqueous phase [33,34]. Furthermore, according to Flegr [33], extraction buffer with ammonium sulphate (AMS) saturation between 20 to 60 % allows ssRNA removal to the phenolic phase. In addition to the phenol-based methods, a total RNA precipitation method has been described, in which DNA remains in solution in the supernatant by adding 0.5 vol ammonium acetate 7.5 M to the total nucleic acid sample [35]. For a cheaper, simpler, and faster dsRNA isolation method we propose an approach based on the solubility of both DNA and ssRNA compared to dsRNA in the phenol phase.

Hereby is presented an optimized method for isolating dsRNA from yeast, as demonstrated in a *Saccharomyces cerevisiae* strain. The approach is a sequential combination and adaptation of the following techniques from previous studies: pre-treatment of *S. cerevisiae* cells with βME [32], the use of pH 4 phenol [33,34] and addition of AMS to remove ssRNA [33] in the extraction method, and the use of ammonium acetate to precipitate total RNA whilst leaving the DNA in the supernatant [35].

The protocol relies on partitioning DNA and ssRNA into the phenolic phase, facilitated by low pH and the presence of AMS in the extraction buffer, respectively, while dsRNA is left in the supernatant. To precipitate the dsRNA from the supernatant, an appropriate volume of ammonium acetate is added to the sample. This new isolation method was thoroughly validated by comparison with the standard fibrous cellulose dsRNA isolation method, followed by an RT-PCR using novel primers targeting the dsRNA L-BC virus from *S. cerevisiae*, and confirmation through Sanger DNA sequencing.

This optimized dsRNA isolation method, which is cheaper, faster, and simpler, has great potential for advancing research in molecular virology and to be applied in dsRNA-based studies in general.

## Materials and methodology

### Reagents and consumables

Tris-hydrochloride (Tris–HCl; VWR Chemicals, cat. no. 33621.260).

Disodium ethylenediamine (EDTA; Merk-Millipore 1.08418).

Sodium dodecyl sulphate (SDS; Carl Roth CN30.2).

2-Mercaptoethanol (βME; Merck-Millipore, cat. no. 805740).

8-Hidroxiquinoline (Sigma 252565).

Absolute Ethanol (Merck-Millipore, cat. no. 1.00983).

Isopropanol (CHEM-LAB CL00.0906)

Phenol (Panreac 141323.1611).

Chloroform (Fisher c/4966/15).

Isoamyl alcohol (Merck-Millipore 100979).

Sodium acetate (NaAc; Merck-Millipore, cat. no. 106268).

Ammonium sulphate (AMS; Merck-Millipore, cat. no. 101217).

Ammonium acetate (Applichem, A3674).

Agarose (Fisher BP-160).

Ethidium bromide (Sigma E-7637).

Cellulose fibers (medium) (Sigma C6288).

Glucose (VWR Chemicals 24379.294).

Yeast extract (Biokar A1202HA).

Peptone (Biokar A1708HA).

1.5 ml microfuge tubes, 15ml, 50 ml falcon-type tubes, 500 ml Erlenmeyer flasks and nuclease-free tips.

### Reagent preparation

#### Solutions

Chloroform:isoamyl alcohol (CIA) 24:1

Ethanol 70 %

TE buffer: 1 mM EDTA (pH 8.0) and 10 mM Tris HCl (pH 7.0)

STE buffer: 1 mM EDTA (pH 8.0) 50 mM Tris HCl 0. 1 M NaCl, (pH 7.0)

TAE buffer: 40 mM Tris-acetate; 1 mM EDTA; (pH 8.0)

Acetate buffer (pH 4): 10 mM NaAc, (pH 4.0)

Acetate buffer (pH 5.6): 2 M NaAc, (pH 5.6)

Ammonium acetate: 7.5 M

100 bp plus DNA Ladder ready-to-use (Bioron #304105)

λ DNA/HindIII Marker (Promega #G1711)

Gel loading buffer BlueJuice TM 6X (Invitrogen, Lithuania)

STE + 16% ethnol buffer: 100 ml STE 10X, 160 ML 100% ethanol, water up to 1 L.

#### Phenol preparation

Preparation of saturated phenol (pH 4 and pH 7):

1. Melt an aliquot of redistilled phenol in a 65 °C water bath or let it melt at room temperature (RT) overnight.
2. Add to the phenol 8-hydroxyquinoline to a final concentration of 0.1 % w/v. Mix to dissolve into both flasks (pH 4 or pH 7).
3. Add an equal volume of distilled sterile water. Mix and allow the phases to separate at RT (this will take approximately 15 to 30 min). Remove the aqueous top layer and discard.
4. Repeat Step 3 now with Acetate buffer (pH 4) or with TE (pH 7) a total of 3 times.
5. Add 0.1 % 2-Mercaptoethanol to the aqueous phase and mix. Allow the phases to separate.
6. The buffer saturated phenol may be stored at 4 °C for periods up to 5 months.

#### Buffers

Extraction Buffer 1: (STE 1x): 100 mM NaCl, 1 mM Na_2_EDTA, 10 mM Tris-HCl, 1 % SDS. Add βME to a final concentration of 2 % (v/v) just before use.

Extraction Buffer 2 (SS20): This is a modified buffer from [32] and with the addition of AMS [33] plus the addition of EDTA and 2-Mercaptoethanol (SS20 buffer): 0,8 % (137mM) NaCl, 10 mM EDTA, 20 %(w/v) (1.5 M) AMS and 0,2 % SDS (pH 4.0). Add βME to a final concentration of 2 % (v/v) just before use.

#### Equipment

Micropipettes for 10 µl, 200 µl and 1000 µl tips.

Thermal cycler.

Spectrophotometer UV-Vis for µl volumes.

Agarose gel electrophoresis chamber.

Gel documentation system.

Laboratory analytical balance.

Refrigerated centrifuge, 15,000 g.

Heat block, for 200 µl or 1,5 ml tubes.

Water bath.

Incubator 37 °C.

Orbital shaker (100 to 300 rpm).

### Yeast culture

The laboratory strain *S. cerevisiae* PYCC 3938 was obtained from PYCC (Portuguese Yeast Culture Collection, FCT-UNL, Portugal). This strain have other collection numbers (CBS 6234, ATCC 46785, CCRC 22576, NRRL Y-11875, NCYC 1026). Accoding to the National Collection of Yeasts CULTURE, STRAIN NCYC 1026 (HTTPS://www.ncyc.co.uk/catalogue/saccharomyces-cerevisiae-1026) has a killer phenotype and possesses a plasmid. This strain was cultivated under aerobic conditions in YPD medium (20 g/L glucose, 10 g/L yeast extract, 20 g/L peptone), under continuous shaking (170 rpm) at 25 °C, for 24 h. Then, 2 % absolute ethanol was added, and stirred continuously for 18 h.

### Cell wall treatment

To avoid the use of enzymes or beads, we used a βME-based method [32] but increased the βME concentration to 5 % and the incubation time to 40 min to ensure efficient cell wall break. After incubation, the cells were collected by centrifugation at 6,000 g/10 min, the supernatant was discharged, and the cell pellet was weighed. The cells were then resuspended in 50 mM Na_2_EDTA (pH 8.0), and pelleted again to remove EDTA. Cell pellets were resuspended and incubated for 40 min in 50 mM Tris-H_2_SO_4_ (pH 9.3) with 5 % βME, and then centrifuged at 10,000 g for 10 min. Cell pellets, of approximately 50 mg, were used immediately or maintained at -80 °C until further use.

### Total nucleic acid extraction

This method [32], with modifications, was used to confirm the total nucleic acids present in the sample and can be used as a first step for the dsRNA isolation methods.

1. Add 500 µl of extraction buffer 1 to 50 mg of pre-treated yeast cells, resuspend the cells and add 500 µl phenol (pH 7) plus 100 µl CIA.
2. Vortex sample for 10 s and incubate at 60 °C for 30 min.
3. Centrifuge at 10,000 g for 10 min.
4. Transfer the supernatant (approximately 500 µl) to a new tube, add 100 µl of CIA and vortex.
5. Centrifuge at 10,000 g for 10 min.
6. Transfer the supernatant to a new tube, add 1 volume isopropanol 100 % and 10 µl Sodium Acetate 2M (pH 5.6)
7. Incubate at -20 °C for 30 min to 1 h.
8. Centrifuge at 10,000 g at 4 °C for 10 min and remove the supernatant.
9. Add 1 ml ethanol 70 % to the pellet, centrifuge at 10,000 g at 4 °C for 1 min and remove the supernatant.
10. Add 500 µl of ethanol 100 %, leave for 1 min, centrifuge at 10,000 g for 2 min and remove the supernatant.
11. Dry the nucleic acid pellet at RT until completely dry and resuspend in 50 µl STE buffer.

### dsRNA isolation – cellulose

This method [18], with modifications, was employed here as a reference method for dsRNA isolation.

1. Add 500 µl of extraction buffer 1 to 50 mg of pre-treated yeast cells, resuspend the cells and add 500 µl phenol (pH 7) plus 100 µl CIA.
2. Vortex sample for 10 seconds and incubate at 60°C for 30 min.
3. Centrifuge at 10,000 g for 10 min.
4. Transfer the supernatant (approximately 550 µl) to a new tube with 75 mg of fibrous cellulose.
5. Add 450 µl of buffer STE 1x plus 200 µl of ethanol 100 %, vortex the sample and incubate for 5 min.
6. Centrifuge at 8000 g for 3 min and discard the supernatant.
7. Add 1ml STE + 16% ethanol buffer to the pellet, centrifuge at 8000 g for 3 min and discard the supernatant.
8. Repeat step 6 and 7 four times.
9. To elute the dsRNA from the cellulose, add 500 µl STE 1x without ethanol to the cellulose pellet, vortex and centrifuge at 8000g for 3 min, collect the supernatant with the eluted dsRNA to a new tube.
10. Add 250 µl STE 1x buffer to the cellulose pellet, vortex and centrifuge at 8000g for 3 min to elute the remaining dsRNA, collect the supernatant and add to the previous one.
11. Add 1 volume isopropanol and 10 µl Sodium Acetate 2M (pH 5.6) to the collected mixed supernatant, incubate at -20 °C for 30 min to 1 h.
12. Centrifuge at 10,000g at 4 °C for 10 min, and remove the supernatant.
13. Add 1 ml ethanol 70 % to the pellet, centrifuge at 10,000g at 4 °C for 10 min, and remove the supernatant.
14. Dry the nucleic acid pellet at RT until completely dry and resuspend in 50 µl STE buffer.

### Improved dsRNA isolation and purification – phase partition

This novel protocol is based on the partitioning of DNA and ssRNA into the phenol phase, facilitated by low pH and the presence of ammonium sulfate in the extraction buffer, respectively. As a result, dsRNA remains in the aqueous phase.

1. Add 500 µl Extraction Buffer 2 (SS20) to a nuclease-free 1.5 ml microcentrifuge tube containing 50 mg pre-treated yeast pellet, and vortex the sample to resuspend the cells.
2. Add 500 µl phenol (pH 4 or pH 7) and 50-100 µl of CIA into the same tube and vortex the mixture for 10 s.

NOTE: The volume of CIA added can be increased to 100 µl if phase separation still does not occur.

3. Incubate at 60 °C for 15 to 20 min in a heat block and invert every 5-10 min to homogenize, then cool to RT.
4. Centrifuge at 14,000 g for 10 min.
5. Transfer the aqueous upper phase to a new tube, then, add 450 µl phenol (pH 4 or pH 7) and 45 µl CIA.
6. Vortex for 5 s to form an emulsion and incubate 15 to 20 min at RT.
7. Centrifuge at 12,000 g for 10 min at RT to separate the organic and aqueous phases.
8. Carefully transfer the aqueous upper phase using a 200 µl micropipette tip into a new tube and add 100 µl CIA.
9. Centrifuge at 10,000 g for 10 min at RT to separate the organic and aqueous phases.
10. Carefully transfer the aqueous upper phase to a new tube and add 1 vol ammonium acetate (7.5M).
11. Gently invert 5–6 times and then incubate at -20 °C for 30 min to precipitate the dsRNA.
12. Centrifuge at 14,000 g for 15 min at 4°C to pellet the precipitated dsRNA.

NOTE: this must be performed at 4°C, to ensure good RNA precipitation.

13. Carefully remove the supernatant without disturbing the pellet and add 500 µl of 70 % (v/v) chilled (-20 °C) ethanol.
14. Centrifuge at 10,000 g for 5 min and discard the supernatant by decanting and add 500 µl 100 % ethanol, to wash the dsRNA pellet from any traces of phenol.
15. Dry at RT and resuspend in 50 µl STE 1 X buffer and maintain the sample at -20°C.

Table 1 summarizes the comparison between the various methods used for dsRNA purification and the alternative method presented in this article.

**Table 1:** Comparison between the various described dsRNA purification methods.

Phases of the method	Methods			
	Total nucleic acids [32]	Cellulose [18]	Flag method [33]	Improved method - phase partition
Cell treatment	Wash with EDTA and treatment with βME	Wash with EDTA and treatment with βME	Enzyme digestion	Wash with EDTA and treatment with βME
Homogenization and RNA extraction	Add STE buffer with SDS	Add STE buffer with SDS	Add SDS lysis protoplasts	Add SS20 buffer
	-	-	Add solid ammonium sulphate.	-
	Phenol: chloroform (5:1) extraction pH 7.0	Phenol: chloroform extraction pH 7.0	Add Phenol extraction pH 4.0	Add Phenol pH 4.0 or pH 7.0, mix by vortex, add chloroform (0,1 vol of the phenol) mix by vortex.
	-	Collect supernatant add 16 % ethanol final concentration.	Centrifuge collect lower phase	Centrifuge collect supernatant
dsRNA purification	(Not tested)	Add cellulose and wash with STE + 16 % ethanol.	-	-
	-	Elute dsRNA with STE buffer.	-	-
dsRNA precipitation	Total nucleic acids precipitation with 1 vol isopropanol	Total nucleic acids precipitation with 1 vol isopropanol	Add 1 vol. isopropanol. Phase separation. Collect low phase and precipitate dsRNA with 60 % ethanol	Add 1 vol. ammonium acetate, incubate for 1 h at 4 °C to -20 °C
	Centrifuge to collect precipitate nucleic acid	Centrifuge to collect precipitate dsRNA	Centrifuge to collect precipitate dsRNA	Centrifuge to collect precipitate dsRNA
dsRNA wash	Pellet wash with ethanol 70 % followed by ethanol 100 %	Pellet wash with ethanol 70 % followed by ethanol 100 %	-	Pellet wash with ethanol 70 % followed by ethanol 100 %

### Qualitative and quantitative analyses of the isolated dsRNA

A simple, rapid, and relatively inexpensive spectrophotometric assay was performed to assess the purity of the extracted dsRNA. Ratios of UV absorption at A260/280 and A260/230 were recorded using Nano-Drop ND-1000 (Thermo-Scientific, USA). Nucleic acid extraction was assessed by agarose gel electrophoresis. DsRNA concentration was determined according to the extinction coefficient of 46 µg/mL/A260 [36,37].

### Validation of RNA quality and integrity using PCR amplification and DNA sequencing

To validate the extracted RNA quality, a fragment from the Viral RNA-directed RNA-polymerase from L-BC Totivirus was amplified using a One-tube RT-PCR [38] with modifications described below.

### Primer design

Forward and reverse primers were designed using Genefisher (https://bibiserv.cebitec.uni-bielefeld.de/genefisher2), an interactive web-based program described by Giegerich et al. [39], using consensus sequence analysis from Viral RNA-directed RNA polymerase from L-BC Totivirus Genbank sequences (NC_001641.1, KX906605.1, KT784813.1). The selected primers (Table 2) were synthesized by Amersham Pharmacia Biotech.

**Table 2:** Primers used in the study.

Name	Sequence (5’-*>*3’)	Amplicon size	position accession number NC_001641
Lev-2-1 forward	TAACAAGAGGGATTGCTACC	650 bp	3894-3913
Lev-2-2 reverse	GCCATTTTGTAGGATCTCCT		4544-4525

### DsRNA pre-treatment before RT-PCR

The dsRNA in STE buffer was diluted with water (1:1) and 1 volume of DMSO 100 % was added (final concentration 50 %). This was denatured at 95 °C for 1 min and then placed on ice [37].

### RT-PCR method

The one-tube RT-PCR validation combines 1st-strand cDNA synthesis by Reverse-transcriptase with the *Taq* DNA polymerase, using the *Taq* polymerase buffer. The validation assay was tested with 3 different PCR buffers:

Buffer 1: 5x, Green GoTaq®Flexi Buffer (Promega Part# M891A): Proprietary formulation supplied at pH 8.5 containing blue dye and yellow dye.

Buffer 2: 10x, *Taq* Fermentas Buffer with KCl (100mM Tris-HCl, pH 8.8 at 25 °C), 500mM KCl,0.8 % Nonidet P40),

Buffer 3: 10x, *Taq* Fermentas Buffer with (NH4)2SO4 (750mM Tris-HCl (pH 8.8 at 25 °C), 200mM (NH4)2SO4, 0.1 % Tween 20.).

Each 25 µl reaction included: reaction 1x Buffer 1, 2 or 3, 1 ng dsRNA, 30 U reverse transcriptase (NZY Reverse Transcriptase # MB12401, NZYTech, Portugal), 0.5 U *Taq* Pol (NZYTaq II DNA polymerase, # MB35402, NZYTech, Portugal), BSA (0.5 mg/ml, NEB #B9001S, USA), 0.1 mM dNTPs (Thermo, USA), 2.5 mM DTT (Sigma 646563), 10 µM each gene-specific primer (Table 1). Amplification conditions were: 39 °C for 90 min, then 5 min at 95 °C, followed by 45 cycles of 1 min at 94 °C, 1 min at 52 °C and 1 min at 72 °C, followed by a final extension step at 72 °C for 10 min carried out in a Sensoquest thermal cycler (Labcycle 48, Germany). The expected size was 650 bp.

NOTES:

1. 2.5 mM DTT final concentration was included to activate the reverse transcriptase because the original buffer for this enzyme (NZY Reverse Transcriptase # MB12401) contained DTT.
2. *Taq* buffers from Fermentas (Lithuania) are no longer commercially available, so the formulations were obtained from the Fermentas catalogue.

### Gel electrophoresis

Ten (10) µl of dsRNA extraction or PCR product were subjected to electrophoresis on a 0.5 mg/ml ethidium bromide (Sigma) stained 1 % (w/v) agarose (Fisher Scientific, USA) gel in TAE buffer. The gels were analyzed and documented on DigiGenius Gel Doc (Syngene, USA).

### DNA sequencing

After electrophoresis at 100 V for 45 min on a 1 % agarose gel in TAE buffer, the band of the desired size was cut from the gel and the DNA was isolated with the MicroElute – Gel Extraction kit (#D6294-01, Omega, USA), according to the manufacturer's instructions. The collected DNA was sequenced by the Sanger method (StabVida, Portugal), using the PCR primers.

## Method validation

**Fig. 1:**
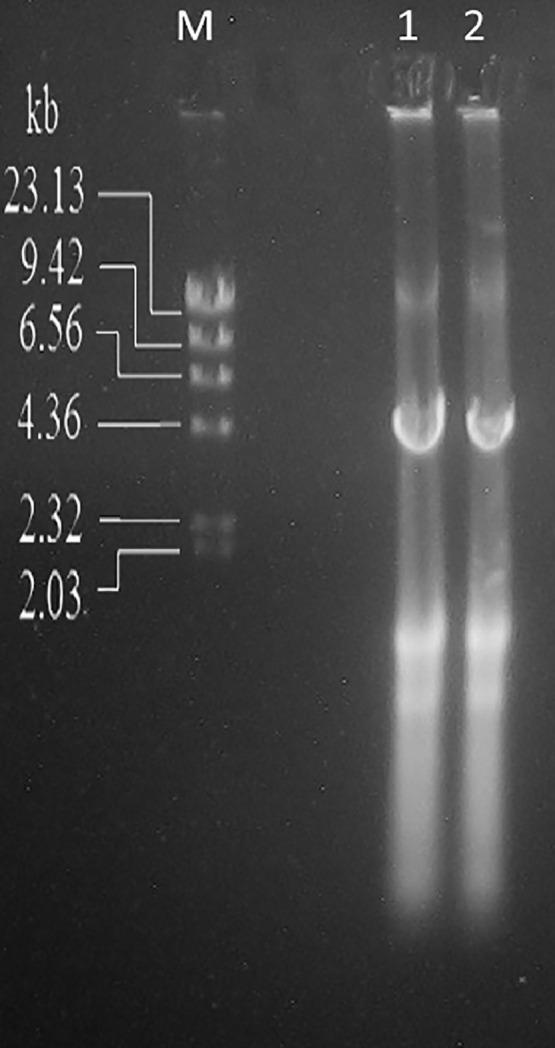
Total nucleic acids purified from *S. cerevisiae* PYCC 3938, using the “Total nucleic acids extraction from yeast” protocol (1 and 2 are duplicate extractions from the same sample). Agarose gel, 1 % in TAE buffer. Marker: Lambda Hind III DNA Marker (Promega).

The yeast cells were treated to break the cell walls using the technique described by Fried and Fink [32], modified by increasing the amount of βME from 2.5 % to 5 % and extending the incubation time from 15 min to 40 min for greater efficiency. Nucleic acids were purified using the "Total nucleic acids extraction from yeast" protocol and include the dsRNA plasmid 4.6 kbp expected band [4,6].The extration was made in duplicate from the same cultured sample 1 and 2 in (Fig. 1).

**Fig. 2:**
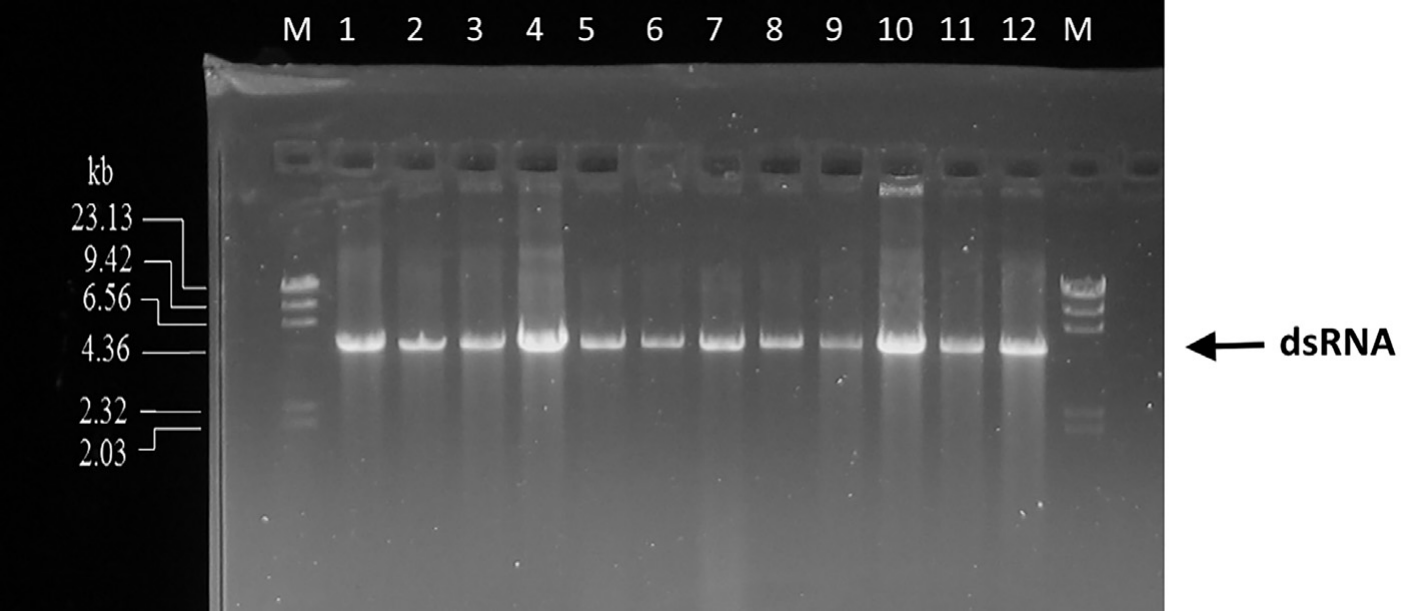
Genomic dsRNA isolation from *S. cerevisiae* PYCC 3938 using dsRNA isolation – phase partition with phenol (initial pH 7) (samples 1, 2, 3 and 4), Cellulose method (5, 6, 7 and 8), and using the dsRNA isolation – phase partition phenol (initial pH 4) (samples 9, 10, 11 and 12). Lane M: lambda HindIII DNA Marker.

To remove both DNA and ssRNA from the nucleic acid solution, we made modifications to the method described by Flegr [33]. The original protocol included the addition of solid AMS to the sample before extraction, but this approach is time-consuming and can be susceptible to contamination. To enhance this protocol, we evaluated incorporation of AMS directly into the extraction buffer. We tested various concentrations of AMS in the extraction buffer (from 5 to 25 % w/v), two phenol pH levels (pH 4 and pH 7), and two incubation temperatures (25°C and 60°C). The optimal AMS concentration was found to be 20 % (w/v), as at greater concentrations dsRNA entered the phenol phase (Supplementary information Fig. 1). However, at 20 % AMS (w/v) the phenol and aqueous phases are inverted, as previously reported by Flegr [33]. To overcome this phenomenon, we added CIA into phenol to a ratio of 10:1. This phenol/CIA mixture, ensured that the aqueous phase remained at the top. We confirmed that 20 % w/v AMS in the buffer separates ssRNA into the phenol phase while preserving dsRNA in the aqueous phase as previously reported by Flegr [33] by gel electrophoresis, as ssRNA become undetectable in the aqueous phase. However, it should be noted that more than two phenol extractions result in dsRNA also being removed into the phenol phase. To effectively remove any DNA that might be present in the sample, the phenol phase should have a low pH. Here, phenol equilibrated with Sodium Acetate at pH 4 [33,34] and phenol at an initial pH 7 produced similar results (Fig. 2), which could be due to the extraction buffer having a final pH of 4, after the addition of AMS, thus reducing the phenol pH. The protocol for dsRNA extraction with best results was: SS20 buffer, 1^st^ phenol:CIA extraction at 60°C, for 30 min and 2^nd^ phenol:CIA at RT. Both ssRNA and DNA were removed from the sample to undetectable levels in gel electrophoresis, but the presence of DNA or ssRNA was not tested by other means.

To precipitate dsRNA, the method described by Flegr [33] was challenging to perform. Instead, we tested adding ammonium acetate solution, which is commonly used for total RNA precipitation [35]. Ammonium acetate volumes from 0.5 vol [35] to 1 vol (3.75 M final concentration) were equally effective at DNA removal, as it remained in the supernatant according to Knapp [35]. However, the higher volume (1 vol) resulted in a higher yield of dsRNA recovered after precipitation (Supplementary information – Fig. 2). We observed no interference from the AMS already present in the buffer, regarding DNA removal or dsRNA yield. This protocol using 1 vol (3.75 M final concentration) ammonium acetate is an efficient and easy to perform alternative method for total RNA precipitation even in the presence of AMS.

The obtained dsRNA yield and purity, using our adapted combined cell wall treatment-extraction-precipitation method, is comparable to the traditional method used for dsRNA purification, which relies on cellulose (Fig. 2 and Table 3)

**Table 3:** DsRNA concentrations obtained with the various methods.

Sample	260/280	260/230 nm	DsRNA concentration (ng/µl)
SS20 buffer phenol pH7	1.88 ± 0.01	1.62 ± 0.34	52,32 ± 2
SS20buffer phenol pH4	1.71 ± 0.15	1.36 ± 0.31	67.35 ± 17
Cellulose purification	2.04± 0.23	1.80 ± 0.39	56.90 ± 14

*Note*: Results are expressed as the mean of 3 samples ± standard deviation.

However, a lower 260/230 ratio was observed after using the SS20 buffer, which could be due to low SDS concentration (0.2 % w/v) used in the extraction buffer.

**Fig. 3:**
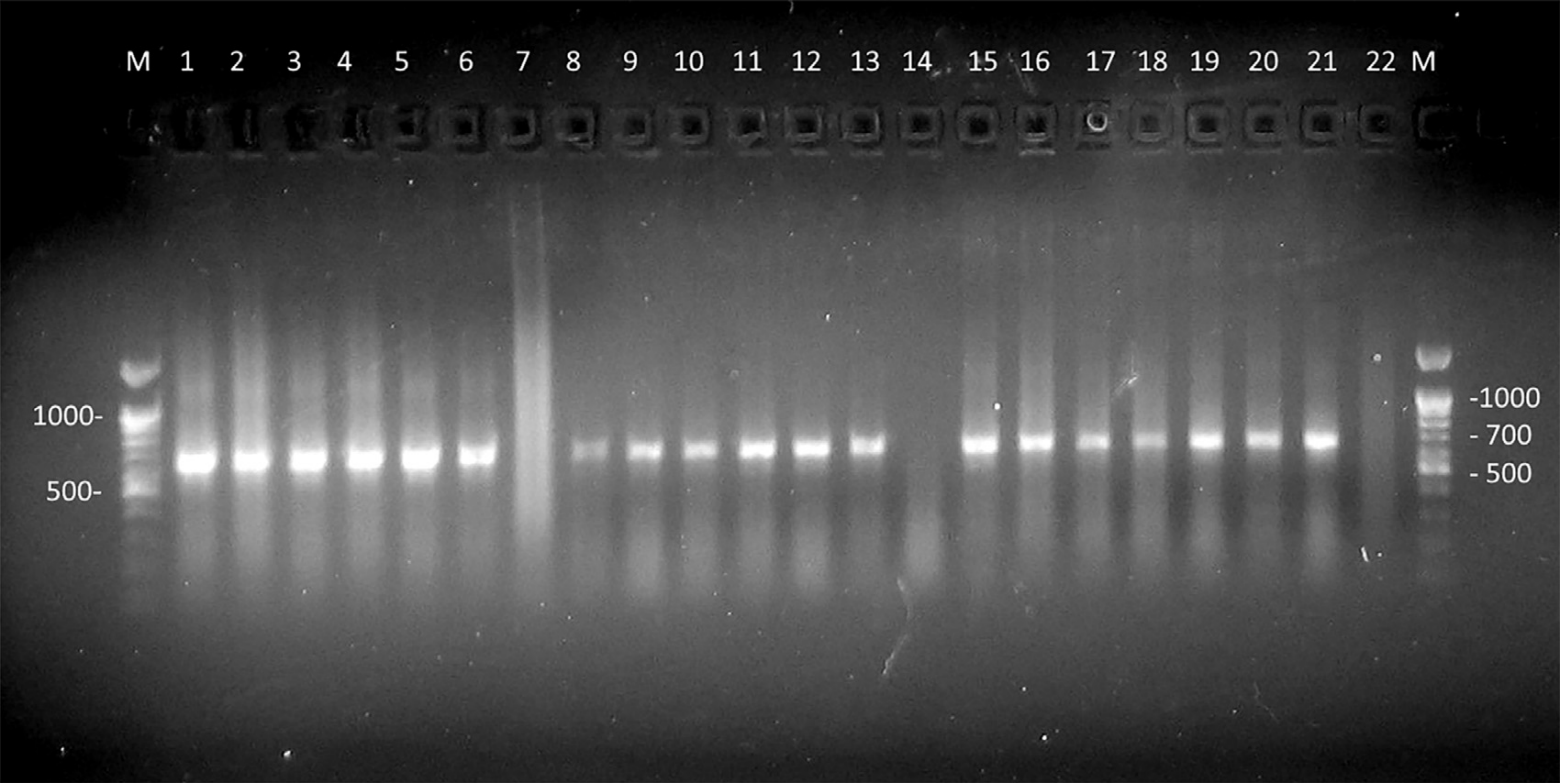
RT-PCR amplification of a 650 bp RNA polymerase gene fragment using dsRNA isolated from *S. cerevisiae*. Lane M: 100 bp DNA Marker (Bioron). Promega buffer (lanes 1, 2, 3, 4, 5, 6, 7), KCL buffer (lanes 8, 9, 10, 11, 12, 13 and 14), NH_4_ buffer (lanes: 15, 16, 17, 18, 19, 20, 21 and 22). PCR mix negative controls samples (lane: 7, 14, 22). dsRNA purification methods: phenol pH 4 (lane 1, 2, 8, 9, 15, 16), phenol pH 7 (lanes 5, 6, 12, 13, 20, 21), Cellulose (lanes 3, 4, 10, 11, 17, 18).

To ensure the presence and quality of the extracted dsRNA, we used one tube RT-PCR amplification of the viral gene RNA-directed RNA-polymerase from L-BC Totivirus, using primers developed by us. For successful amplification, we found that a prior dsRNA denaturation step with DMSO at 95 °C for 1 min was necessary before adding it to the RT-PCR mix. Without this step, or with different temperatures and incubation times (37°C for 30 min or 65°C for 20 min), no RT-PCR amplification was observed. The dsRNA samples isolated using the different methods, including the new phenol partition method with an AMS buffer, were equally amenable to successful RT-PCR amplification with L21/L22 primers, producing the expected 650 bp amplicon with similar band intensity (Fig. 3).

The amplicon was sequenced using Sanger sequencing, yielding a high-quality sequence (Supplementary information- Fig. 3). Upon a BLASTn search against the National Center for Biotechnology Information (NCBI) database, this sequence was found to have 97.64 % similarity to the previously reported *S. cerevisiae* L-BC virus sequence (accession NC_001641.1), and a BLASTp search gave 95.44 % similarity with *S. cerevisiae* PYCC 3938 strain that harbors Totivirus L-BC-2 virus (accession APR62628.1). The cDNA sequence has been deposited in GenBank under accession number OR393309.

We describe, here, a new method for dsRNA extraction and purification that, in summary, combines an initial cell wall treatment with 5 % βME, AMS at 20 % in the extraction buffer for the phenol:CIA (10:1) extraction step and ammonium acetate (1vol 7.5 M) for dsRNA precipitation.

The methods described by Fleg [33] for dsRNA isolation primarily focus on low pH buffer and on the use of ammonium sulfate added to the extraction buffer after an initial extraction, and they are specifically applied to *Trichomonas sp.* dsRNA isolation. Our method, dsRNA isolation – phase partition, offers a more practical and applicable approach to isolating dsRNA in *S. cerevisiae*. It allows the removal of both DNA and ssRNA into the phenol phase using a single buffer, facilitated by maintaining a low pH (4) and incorporating AMS in the aqueous phase.

Another improvement in our method was the addition of chloroform to the extraction buffer, which facilitated dsRNA extraction and prevented the inversion of the aqueous/phenol phases due to the increased density resulting from the addition of ammonium sulfate in the buffer.

Moreover, our method yields similar or even better results in terms of the quantity of isolated dsRNA when compared to the traditional cellulose-based dsRNA methods [15], [16], [17], [18], [19], [20], [21]. Despite lower 260/230 nm rations, the dsRNA obtained was of sufficiently good quality and quantity for RT-PCR amplification. In relation to the cellulose method of dsRNA purification, our method offers several advantages. It reduces the number of extraction and purification steps and cuts down the purification time from 4  h to 2  h.

We not only were able to isolate high-quality genomic dsRNA in yeast, but we also demonstrated that it is well-suited for amplification followed by sequencing. Further tests of this method will include direct RNA sequencing applications.

## Conclusion

The improved method for genomic viral dsRNA isolation presented here addresses a crucial aspect of virology research, by offering a practical and efficient approach to sample preparation, suitable for various molecular analytical techniques and dsRNA amplification, including the detection and discovery of dsRNA virus in pathogens and their hosts (work in progress).

Our approach stands out from the numerous methods published for dsRNA extraction, for its low cost, simplicity, reliability, and reproducibility, making the extraction of high-quality dsRNA from yeast samples more available for low-technology laboratories.

## Ethics statements

We employed yeast for our experiments and adhered to institutional safety and laboratory guidelines for yeast handling.

## CRediT author statement

**Fernando M.H. Cardoso**: manuscript, writing, conceptualization, methodology, preliminary assays of all methods, laboratory work and method optimization. **Alexandre Elias and Inês Pereira**: laboratory work and method optimization. **Isabel Mauricio** manuscript revision and **Olga Matos** writing-review and editing.

All authors revised and approved the final manuscript.

## Declaration of Competing Interest

The authors declare that they have no known competing financial interests or personal relationships that could have appeared to influence the work reported in this paper.

## Data Availability

Data will be made available on request.

## Appendix Supplementary materials

Image, application 1
