# Supplementary material for: Improved dsRNA isolation and purification method validated by viral dsRNA detection using novel primers in Saccharomyces cerevisiae
Source: MethodsX. 2023 Oct 11;11:102435. doi: 10.1016/j.mex.2023.102435 (PMC10591000; doi:10.1016/j.mex.2023.102435)
Supplement: Supplementary file 1 [file mmc1.docx]

***Supplementary material***

*
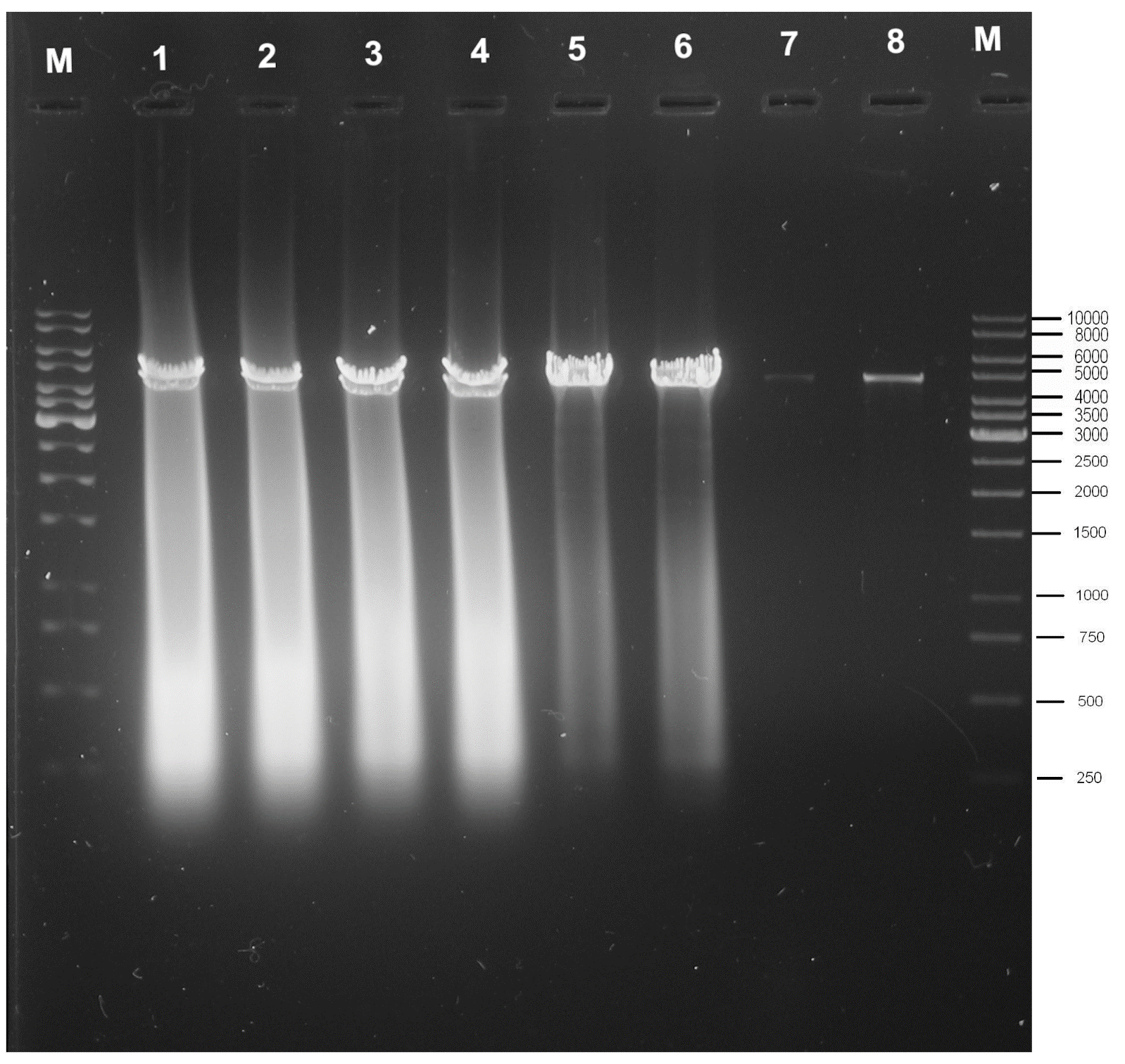
*

**Supplement – fig. 1**. Electrophoresis on a 1% agarose gel, stained with ethidium bromide, of dsRNA from S. cerevisiae PYCC 3938, extracted with buffer (137mM NaCl and 10 mM EDTA) at different concentrations of (NH4)2SO4 (w/v) and with one phenol extraction (pH 4). Lanes 1 – 8: 5% 7.5%, 10%, 12.5%, 15%, 20%, 22.5% and 25%, respectively. M: 1 kb DNA Ladder Plus (MBI – Fermentas). marker size in bp).

*
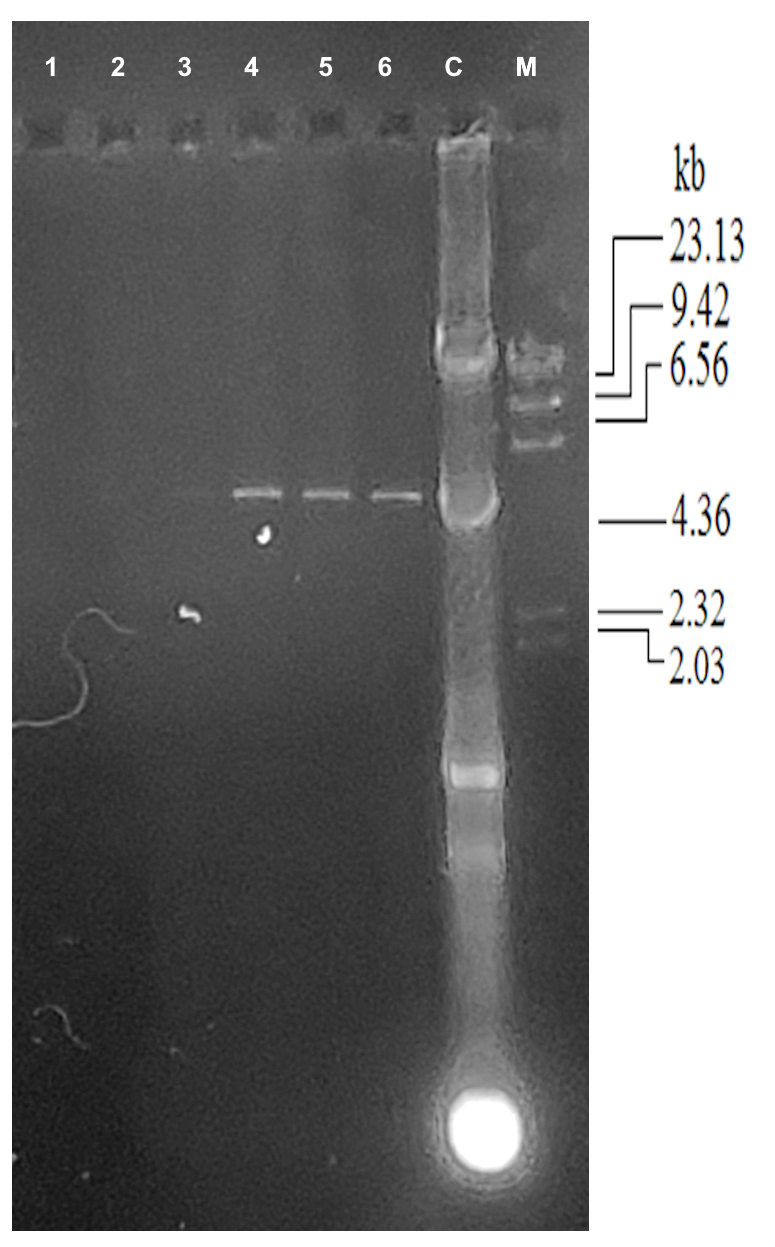
*

**Supplement – fig. 2.** Electrophoresis on a 1% agarose gel, stained with ethidium bromide, of dsRNA from S. cerevisiae PYCC 3938, precipitated with different volumes of Ammonium Acetate (7.5 M initial concentration). Lanes 1 – 6: 1: 0.25 vol, 2: 0,5 vol, 3:0,75 vol, 4:1,0 vol, 5:1,25vol, 6:1,5 vol, C: total nucleic acids from S. cerevisiae PYCC 3938, M: Lambda Hind III marker size in bp).

*
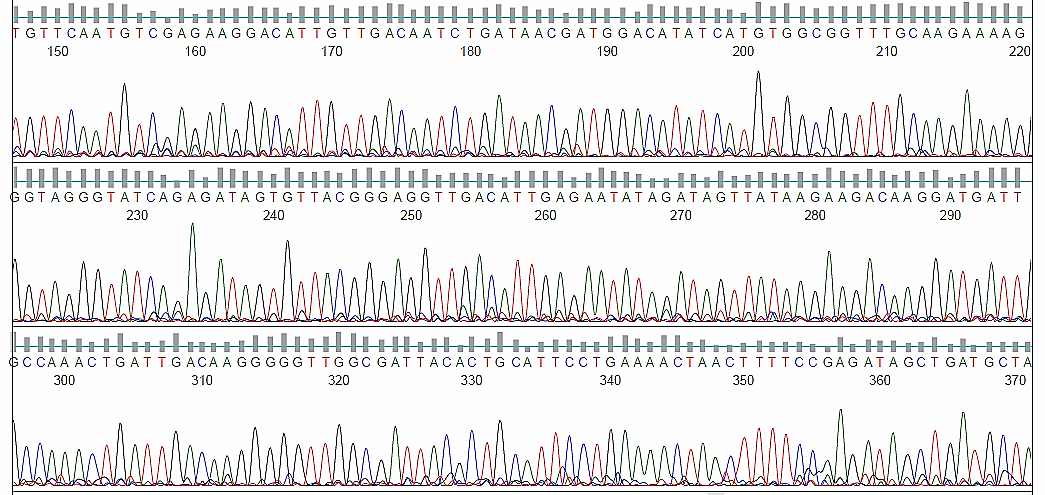
*

**Supplement – fig. 3.** Chromatogram of a fragment of the Viral Rna polymerase from Totivirus , L-BC virus gene.
